# Approach to a Patient with Urosepsis

**DOI:** 10.4103/0974-777X.52984

**Published:** 2009

**Authors:** Om Prakash Kalra, Alpana Raizada

**Affiliations:** *Division of Nephrology, University College of Medical Sciences and G.T.B. Hospital, Dilshad Garden, Delhi, India*

**Keywords:** Pyelonephritis, Urinary tract infection, Urosepsis

## Abstract

Urinary tract infections can occur in all age groups and produce an exceptionally broad range of clinical syndromes ranging from asymptomatic bacteriuria to acute pyelonephritis with Gram negative sepsis to septic shock. In approximately one-quarter of all patients with sepsis, the focus of infection is localized to the urogenital tract. This may lead to substantial morbidity and significant economic implications. We present a review of the current approaches to managing urospesis.

## INTRODUCTION

Urosepsis implies clinically evident severe infection of the urinary tract and/or the male genital tract (e.g. prostate) with features consistent with systemic inflammatory response syndrome.[1] It may be associated with multi-organ dysfunction, hypo-perfusion or hypo-tension. Severe sepsis is usually associated with pulmonary and abdominal infections with urinary tract infections (UTIs) accounting for about five per cent cases.[2] However, among the nosocomial infections, UTIs account for approximately 40% of the cases.[3,4] Though sepsis is commoner in men than in women, it has been found that urosepsis is commoner in women than in men. While severe sepsis has a reported mortality rate of 20 to 42%, urosepsis may be associated with high mortality rates in special patient groups.[5] Therefore, patients with urosepsis should be identified at an early stage and promptly treated to prevent development of organ failure and other complications.

Complicated UTI is the commonest precursor of urosepsis. Complicated UTI usually refers to an infection that occurs in a patient with a structural or functional abnormality, impeding urine flow, or in a host with altered defences or in patients with metabolic disorders like diabetes or azotemia. When complicating factors are present, antimicrobial resistance is more common and the response to therapy is often disappointing, even with agents active against the causative microbial pathogens. In addition, severe complications frequently occur which may lead to urosepsis, renal scarring or even end-stage renal disease. Drug treatment of urosepsis often has to be complemented with endoscopic and/or surgical intervention.

## RISK FACTORS

Several genito-urinary abnormalities may be associated with urosepsis. The most common determinant of urosepsis is obstruction to free flow of urine which may quickly lead to severe sepsis. Various structural and functional abnormalities of the genito-urinary tract associated with urosepsis have been mentioned in Table 1. Patients at higher risk for developing urosepsis include elderly patients, diabetics and immuno-suppressed patients such as transplant recipients, patients with AIDS, patients receiving anticancer drugs and immunosuppressive agents. Any male urinary infection is usually considered complicated as uncomplicated urinary infection is rare in men.[6] Bacteriuria is more prevalent among the elderly, with 50% of geriatric women having bacteriuria. The risk of infection varies with different abnormalities.

**Table 1:** Structural and functional abnormalities of the genitourinary tract associated with urosepsis

Obstruction
Congenital: Ureteric or urethral strictures, phimosis, ureterocele, polycystic kidney disease
Acquired: Calculi, prostatic hypertrophy, tumors of the urinary tract, trauma, pregnancy, radiation therapy
Instrumentation
Indwelling urethral catheter, ureteric stent, nephrostomy tube, urological procedures
Impaired voiding
Neurogenic bladder, cystocele, vesicoureteral reflux
Metabolic abnormalities
Nephrocalcinosis, diabetes, azotemia
Immundeficiencies
Patients on immunosuppressive drugs, neutropenics

The risks are multiplied in cases of hospitalization or long- term care due to indwelling long-term catheters and the transfer of resistant bacterial strains. In a study conducted during 1990s, Richards *et al.*, noted that 23% of all cases of hospital-acquired sepsis were due to UTI and mostly seen in catheterized patients.[7]

ESGNI-004, a pan-European study, reported an incidence of hospital-acquired UTIs of 3.55/1,000 patient days and 51.5% of all patients were febrile, 31.9% went on to develop plain sepsis, 2% severe sepsis, 0.3% septic shock and 1.7% multi-organ failure.[8] Two point-prevalence studies about hospital-acquired UTIs, the Pan European Prevalence (PEP) study and the Pan EuroAsian Prevalence (PEAP) study were carried out during the year 2003–2004. The prevalence of hospital-acquired UTIs in the PEP study was 10% and 14% in the PEAP and urosepsis accounted for 12% of all episodes.[4]

## MICROBIOLOGICAL DATA

Gram-negative bacilli account for majority of the cases of urosepsis. These include *Escherichia coli* (50%), *Proteus* spp. (15%), *Enterobacter* and *Klebsiella* spp. (15%), and *Pseudomonas aeruginosa* (5%) which dominate the bacterial spectrum in urosepsis, while Gram-positive organisms are involved less frequently (15%).[9] The ESGNI-004 reported that Gram-positive organisms represented 21.2% of all hospital-acquired UTI isolates, whereas Gram-negative organisms accounted for 65.9% and yeasts 12.9%. The ESGNI-004 found that in the catheterized patients *Candida* spp. and *P. aeruginosa* were more common, with *E. coli* being the commonest bacterium isolated in both catheterized and non-catheterized patients. Data from the PEP and PEAP studies reported a microbiologically proven infection in 74% of patients (urine culture 91%, blood culture 7%, other source 2%). In another multi-centre, prospective study from Portugal, 7% patients admitted to Portuguese ICUs had urosepsis with isolation rate of 68% and blood culture positivity of 41%.[10] All isolates except one were Gram-negative rods with *E. coli* dominating the microbiological profile.

*E. coli* strains isolated from symptomatic patients with complicated urinary infection have a lower prevalence of genetic or phenotypic virulence characteristics and are less likely to originate from a uropathogenic clone than strains isolated from patients with acute uncomplicated infection. Polymicrobial bacteriuria is seen in elderly patients and patients with chronic urological devices. The micro-organisms commonly responsible for post-transplant urosepsis are the enteric Gram-negative bacilli and enterococci. Organisms isolated from patients with complicated urinary infection and urosepsis tend to be more resistant to antimicrobials than strains isolated from uncomplicated urinary infection.

## CLINICAL PRESENTATION

The clinical presentation may be varied. Urosepsis represents the most severe clinical manifestation of UTI. Signs and symptoms of systemic inflammatory response syndrome such as fever, tachycardia, tachypnea, respiratory alkalosis which were earlier considered mandatory for the diagnosis of sepsis, are now considered to be the alerting symptoms. These have been mentioned in Table 2.

**Table 2:** Systemic inflammatory response syndrome

Body temperature ≥ 38°C or ≤ 36°C
Tachycardia ≥ 90 beats/min
Tachypnea ≥ 20 breaths/min
Respiratory alkalosis PaCO_2_ ≤ 32 mm Hg
Leucocytes ≥ 12 000 cells/μL or ≤ 4000 cells/μL or bandforms > 10%

Only one-third patients classically present with fever and chills along with hypotension. The patient may present with tachypnea, tachycardia, and can have a febrile flushed appearance with alteration of mental status. Initially, because of increased cardiac output and decreased systemic vascular resistance, the clinical appearance is that of ‘warm shock’ with hypotension but with further loss of intravascular volume and decreased vascular tone the manifestation is that of ‘cold shock’ with hypotension and cold extremities. This is an ominous development signifying need for prompt intervention. Additional symptoms like flank pain, renal angle tenderness, ureteric or renal colic, dysuria may be present.

With further progression there may be appearance of pulmonary edema with ARDS. Multi-organ failure may follow with renal and/or hepatic dysfunction and disseminated intravascular coagulation.

## EVALUATION

History is crucial in the evaluation of any UTI. It should include any previous history of infections, antibiotic use, and a timeline of symptoms. If possible, any laboratory results associated with previous infections, including culture results should be obtained. The physician should promptly look for evidence of sepsis. A patient can be considered to have evidence of sepsis if he has evidence of bacteremia or clinical suspicion of sepsis accompanied by greater than or equal to two criteria of systemic inflammatory response syndrome as mentioned in Table 2. A thorough physical examination (including a pelvic examination and digital rectal examination to exclude acute prostatitis) should also be performed. If suspicion arises, then further studies may be considered after treatment and recovery from acute illness. Blood culture and urine culture are mandatory investigations during the initial evaluation.

### Urine culture

The diagnosis of UTI, from simple cystitis to complicated pyelonephritis with sepsis, can be established with absolute certainty only by quantitative cultures of urine. Urine specimen must be cultured promptly within two hours after collection or be preserved by refrigeration. A positive urine culture confirms, but is not diagnostic of, symptomatic urinary infection. A negative urine culture, however, has a high negative predictive value, and is useful for excluding urinary infection. The profile of antibiotic resistance rates has not been specifically described in detail for patients with urosepsis, but it is reasonable to assume that the resistance pattern is likely to be similar. In SENTRY Antimicrobial Surveillance Programme, carried out from 1997–1999, isolation of *E. coli* from hospitalized patients showed cotrimoxazole resistance in nearly 25% cases.[11–13]

### Blood culture

Some patients with severe urosepsis may develop bacteremia. In the PEP and PEAP studies, microbiologically proven infection was found in 74% of the patients. Of these, 7% were found to have positive blood culture.[14] A Portuguese study reported an isolation rate of 68% with 41% positive blood cultures.[10] The specimens must be taken before starting empirical antibiotic therapy.

### Localization of underlying abnormality

Whenever complicated urinary infection and urosepsis are suspected but urological abnormalities have not been defined, various special investigations may be carried out. Plain abdominal radiograph is of limited value except that it shows the presence and extent of calcification and calculi within the kidney or urinary tract. It is of help in monitoring change in position and increase in size or number of renal stones. Intravenous urography provides anatomical details of the calyces, pelvis and ureter which are important for the diagnosis of reflux nephropathy and papillary necrosis. However, in a critically ill patient with urosepsis, ultrasonography, CT scan and MRI have been found to be more useful.

#### Ultrasonography:

Ultrasound scan is especially valuable for emergency imaging in patients presenting with first episode of infection with severe loin pain and fever. While it can define kidney size and identify renal scars, it gives little anatomical detail of the pelvicalyceal system or ureters. It is helpful in evaluation of prostate gland and various complications of acute pyelonephritis such as emphysematous pyelonephritis, renal abscess and perirenal abscess.

#### CT scanning and MRI:

These are very useful investigations in patients with urosepsis. These are the most precise methods for identifying bacterial interstitial nephritis and micro-abscesses within the kidney, perinephric abscesses, emphysematous pyelonephritis and renal papillary necrosis.

## GENERAL MANAGEMENT OF SEPSIS AND SEPTIC SHOCK

Urosepsis denotes the most severe and dreaded form of UTI and therefore its management is significantly different from that of simple UTI because, besides antibacterial therapy adjuncts like volume expansion, euglycemia maintenance, inotropic and ventilatory support, etc. are also required. Prompt intervention should be made to meet the following goals:

### Hemodynamic support:

Adequate organ perfusion must be ensured. Hypotension should be managed initially with intravenous fluid administration and the goal should be maintenanace of pulmonary capillary wedge pressure at 12-16 mm Hg or central venous pressure at 8-12 cm H_2_O. Urine output rate should be kept at greater than 0.5 mL/kg/hr. A mean arterial blood pressure of greater than 65 mmHg (systolic blood pressure greater than 90 mmHg) and a cardiac index of greater than or equal to 4 L/min/ m^2^ should be maintained. Vasopressor therapy should be initiated in the event of failure to achieve these goals with iv fluids alone. These include dopamine, dobutamine and norepinephrine.

### Respiratory support:

Ventilatory support should be provided for patients with progressive hypoxemia, hypercapnea, altered sensorium or respiratory muscle fatigue. A study of ‘early goal directed therapy’ (EGDT) found that prompt resuscitation to maintain SvO_2_ >70% was associated with improved survival in patients of severe sepsis.[15] In this study, failure to maintain saturation after fluids and vasopressors was followed by erythrocyte infusion to raise hematocrit to 30%. Patients requiring mechanical ventilation should be adequately sedated and stress ulcer prophylaxis should be administered.

### Metabolic support:

Blood glucose levels should be maintained at less than 150 mg/dL during initial few days of severe sepsis and normoglycemic range could be targeted later. Frequent blood glucose monitoring should be done to avoid hypoglycemia in patients on intensive insulin therapy.

Multi-organ dysfunction, if any should be managed. Disseminated intravascular coagulation, if accompanied by major bleeding, should be treated with fresh-frozen plasma and platelet transfusion. Hypercatabolic individuals with acute renal failure benefit substantially from hemodialysis or hemofiltration. Prophylaxis for deep vein thrombosis and nutritional supplementation should be undertaken.

## ANTIMICROBIAL THERAPY

Empirical antimicrobial therapy effective against both Gram- positive and Gram-negative bacteria should be initiated. The antimicrobial choice should be reassessed once culture results become available, usually within 48 to 72hours. Maximum recommended doses of antibiotics should be administered intravenously with dose/dose interval modifications as required for renal and hepatic failure. Prompt treatment with effective antimicrobial therapy ensures better outcome in septic shock.[16,17] Inappropriate antimicrobial therapy in severe UTI is associated with higher mortality rate.[18] Therefore, choice of empirical antibiotic therapy should be based on the underlying lesion, the most likely causative organism and the local prevalence of resistance pattern.[19] In a retrospective cohort study, it was found that administration of an effective antimicrobial within the first hour of documented hypotension was associated with a survival rate of 80%.[20] Each hour of delay in the subsequent six hours was associated with an average decrease in survival by eight per cent.[20]

*E.coli* and other Enterobacteriaceae are the commonly isolated organisms in patients with community-acquired primary urosepsis. In such cases a third generation cephalosporin, piperacillin in combination with a beta-lactamase inhibitor or a fluoroquinolone with propensity to achieve high urinary concentration (e.g. ciprofloxacin, levofloxacin) should be used for empirical therapy. A combination therapy with an aminoglycoside or a carbapenem may be essential in areas with high rate of fluoroquinolone resistance. For hospital-acquired urosepsis especially urosepsis following urological interventions, an antipseudomonal third-generation cephalosporin or piperacillin/beta-lactamase inhibitor in combination with an aminoglycoside or a carbapenem may be initiated as empirical therapy. Combination antimicrobial therapy has not been found to be superior to monotherapy except that aminoglycoside monotherapy for *P. aeruginosa* bacteremia is less effective than the combination of an aminoglycoside with an antipseudomonal beta-lactamase inhibitor agent.[21]

### Duration of therapy

Most patients require treatment for about 14-21 days. Successful antimicrobial therapy will usually ameliorate symptoms promptly, with substantial clinical improvement in 48 to 72 hours. Patients who fail to respond in this time frame should be reassessed to exclude urinary obstruction or abscess (which may require drainage), to exclude resistance of the infecting organism to the antimicrobial agents, or to consider an alternate diagnosis other than urinary infection. If a complicating factor mandating treatment is identified then removal/control of the factor should follow immediately. Follow-up cultures should be done 2-4 weeks after cessation of therapy to confirm cure.

## SPECIAL CONSIDERATIONS

### Urinary infections in diabetes mellitus

Patients with diabetes mellitus are at a great risk of developing various complications of UTI including sepsis. The predisposing factors are poor glycemic control, presence of autonomic neuropathy, high urinary glucose, impairment in immune function, diabetic cystopathy, diabetic microangiopathy, large-vessel renal vascular disease and increased bacterial adherence to uroepithelial cells. Various characteristic features of UTI in patients with diabetes mellitus have been mentioned in Table 3.

**Table 3:** Characteristic features of urinary tract infections in diabetes mellitus

Increased risk of asymptomatic bacteriuria
Higher risk of urosepsis
Higher risk of recurrent infections
Bilateral infections are more common
Nosocomial infections are more common
Greater likelihood of antimicrobial resistance
Fungal urinary tract infections is more common
Increased risk of complications like renal failure and septicemia
Higher prevalence of atypical uropathogens
Certain infections occur almost exclusively in diabetes mellitus such as emphysematous pyelonephritis, renal papillary necrosis, prostatic abscess

#### Acute pyelonephritis:

The clinical presentation of acute pyelonephritis in diabetic patients is similar to non-diabetics except that bilateral infection is more common in diabetics. Treatment includes hydration and parenteral antibiotics. A poor response to antibiotics suggests complications like emphysematous pyelonephritis, papillary necrosis, perinephric and renal abscess.

#### Emphysematous pyelonephritis:

It is a rare necrotizing renal parenchymal and perirenal infection caused by lactose fermenting bacteria that occurs primarily in diabetics with or without urinary tract obstruction. It is characterized by gas formation within the renal parenchyma, collecting system and/ or perirenal space.[22] Emphysematous pyelonephritis mainly affects females and is associated with high mortality rate if not treated adequately. The main causative organisms are *E. coli* (68%), followed by *Klebsiella pneumonia, Proteus, Pseudomonas, Citrobacter* and rarely *Candida* and *Cryptococcus*. Multiple infections are present in 14-20% cases and positive blood cultures are seen in 54% cases. Many of these patients can be salvaged by broad spectrum antibiotics and percutaneous drainage. In the event of lack of response, nephrectomy may be warranted.

#### Renal abscess:

Over 50% patients with renal abscess have diabetes. High intravesical pressure causing reflux of infected urine has been classically described as the pathogenetic factor of renal abscess in diabetic patients. *E. coli* (75%) followed by *Klebsiella* and *Proteus* (15–20%) are the causative micro-organisms. Most patients have a history of recurrent UTI, renal calculi, and/or prior genitourinary instrumentation. The diagnosis is established by imaging studies. Treatment comprises of broad spectrum antibiotics and percutaneous drainage.

#### Perinephric abscess:

A perinephric abscess is a collection of suppurative material in the perinephric space. It is difficult to diagnose clinically and a high index of suspicion is required in susceptible patients. *E. coli, Proteus spp., and Staphylococcus aureus* are the common causative organisms. It may present one to two weeks after history of UTI with fever and flank pain. The mainstay of treatment for perinephric abscess is drainage. When kidneys are not functioning or are severely infected, nephrectomy may be required.

#### Renal papillary necrosis:

Renal papillary necrosis refers to ischemic necrobiosis of the papilla in the medulla of the kidneys. Diabetes mellitus is the most common predisposing factor as about 59% of cases of renal papillary necrosis occur in diabetics. Passage of sloughed papillae can cause renal colic, ureteric obstruction and rarely urinoma. Rarely renal papillary necrosis can present as acute oliguric renal failure. On intravenous pyelogram ‘moth eaten appearance’ and ‘ring shadows’ are characteristic appearances of renal papillary necrosis. Avoidance of NSAIDs, prompt glycemic control and prompt treatment of pyelitis constitute the treatment for renal papillary necrosis.

#### Prostatic abscess:

Although acute and chronic prostatitis are not found with a higher frequency in diabetics, prostatic abscess is found almost exclusively in diabetic patients and immunosuppressed patients. Rarely the prostatic abscess may become giant-sized and may even rupture into the urethra.[23] The organisms most frequently involved are *E. coli* and *Staphylococcus*. Symptoms are similar to acute prostatitis. Transrectal sonography, CT and MR imaging may aid in diagnosis. The treatment of prostatic abscess consists of transrectal ultrasound guided drainage with appropriate antibiotic therapy.

### Fungal urinary infections

Factors pre-disposing to fungal urinary tract infections are diabetes mellitus, indwelling urethral catheters or other urological devices, pancytopenia, prolonged broad-spectrum antimicrobial therapy. *Candida albicans* is the most common isolate followed by *Candida glabrata*, the isolation of which may be increasing with the widespread use of azoles, to which this species is less susceptible.[24] In a few cases it may lead to fungemia and shock. Catheter removal leads to spontaneous resolution of most episodes of candiduria associated with indwelling catheters. However, in patients with features of sepsis, systemic antifungal therapy is required. Both azoles and amphotericin B are effective for the treatment of symptomatic fungal urinary infection. *Candida* species may have increased resistance to azoles, and systemic amphotericin B may be necessary to treat infection due to some of these species.

### UTI in renal transplant recipients

In renal transplant recipients UTI is the most common form of bacterial infection. Renal transplant recipients are at a higher risk of developing sepsis because of immunosuppressive therapy. Prompt diagnosis of UTI is necessary as unrecognized bacterial infection may even lead to graft loss. The micro-organisms commonly responsible for post-transplant UTI are the enteric Gram-negative bacilli and enterococci. *Corynebacterium urealyticum* (group D2) has been recognized as a potential new pathogen and it may be responsible for as many as 10% cases (vs less than two per cent of UTI in the general population).[25] This has clinical implications as *Corynebacterium urealyticum* is difficult to isolate and is not sensitive to conventional oral antibiotics. The antimicrobial of choice is vancomycin.

Pyelonephritis of the graft is a serious complication of UTI, especially in the transplanted diabetic. UTI may be complicated by papillary necrosis and can provoke rejection episodes. In some cases it may even lead to emphysematous pyelonephritis of the transplanted kidney.[26] Therefore, in the early post-transplant period even low-count bacteriuria and asymptomatic bacteriuria should be treated for a sufficient period of time. This can also be extended beyond the early post-transplant period. Recurrent UTI is frequent in the renal transplant recipient. Relapse usually indicates administration of antibiotics for shorter than 3 weeks. Patients with frequent episodes of re-infection should be investigated to exclude urological abnormalities or neurogenic bladder. It should be remembered that native kidneys may be the source of UTI, particularly when vesicoureteric reflux is demonstrable into the native ureter. DMSA scintigraphy with tomography is useful in the detection of renal scars in transplant kidney. Graft biopsy is superior in evaluation of deteriorating graft function. In patients with normal urinary tract prophylactic treatment for at least 1 year is advised. Cystoscopy is indicated in patients with recurrent UTI to exclude uroepithelial carcinoma, especially in analgesic abusers.

## PREVENTION

The major strategy to prevent urosepsis involves characterizing and correcting the underlying genitourinary abnormalities that promote infection. Wherever correction is not possible, patients with persistent abnormalities remain at risk for recurrent infection and urosepsis. Currently, there are no adult populations at risk for recurrent complicated urinary infection in whom long-term prophylaxis to prevent urinary infection is routinely recommended. Future developments in catheter biomaterial to inhibit biofilm formation may limit biofilm-associated infections. Indwelling urethral catheters for bladder drainage should be used only when clear clinical indications exist, should be inserted using sterile aseptic technique, be maintained with a closed drainage system, and should be removed as soon as clinically feasible. Catheters should be replaced before initiating antimicrobial therapy for the treatment of a symptomatic episode.

## CONCLUSION

Urosepsis is a dreaded complication of UTI with mortality rates as high as 40%. Early recognition of symptoms followed by appropriate investigations, accurate diagnosis and early goal directed therapy is essential to improve outcomes. Comprehensive management requires team approach with timely inputs from microbiologists, radiologists, surgeons and intensive care physicians.
